# VESPA: software to facilitate genomic annotation of prokaryotic organisms through integration of proteomic and transcriptomic data

**DOI:** 10.1186/1471-2164-13-131

**Published:** 2012-04-05

**Authors:** Elena S Peterson, Lee Ann McCue, Alexandra C Schrimpe-Rutledge, Jeffrey L Jensen, Hyunjoo Walker, Markus A Kobold, Samantha R Webb, Samuel H Payne, Charles Ansong, Joshua N Adkins, William R Cannon, Bobbie-Jo M Webb-Robertson

**Affiliations:** 1 Scientific Data Management, Pacific Northwest National Laboratory, Richland, WA, USA; 2 Computational Biology and Bioinformatics, Pacific Northwest National Laboratory, Richland, WA, USA; 3 Biological Separations and Mass Spectrometry, Pacific Northwest National Laboratory, Richland, WA, USA; 4 Software Systems and Architecture, Pacific Northwest National Laboratory, Richland, WA, USA

## Abstract

**Background:**

The procedural aspects of genome sequencing and assembly have become relatively inexpensive, yet the full, accurate structural annotation of these genomes remains a challenge. Next-generation sequencing transcriptomics (RNA-Seq), global microarrays, and tandem mass spectrometry (MS/MS)-based proteomics have demonstrated immense value to genome curators as individual sources of information, however, integrating these data types to validate and improve structural annotation remains a major challenge. Current visual and statistical analytic tools are focused on a single data type, or existing software tools are retrofitted to analyze new data forms. We present Visual Exploration and Statistics to Promote Annotation (VESPA) is a new interactive visual analysis software tool focused on assisting scientists with the annotation of prokaryotic genomes though the integration of proteomics and transcriptomics data with current genome location coordinates.

**Results:**

VESPA is a desktop Java™ application that integrates high-throughput proteomics data (peptide-centric) and transcriptomics (probe or RNA-Seq) data into a genomic context, all of which can be visualized at three levels of genomic resolution. Data is interrogated via searches linked to the genome visualizations to find regions with high likelihood of mis-annotation. Search results are linked to exports for further validation outside of VESPA or potential coding-regions can be analyzed concurrently with the software through interaction with BLAST. VESPA is demonstrated on two use cases (*Yersinia pestis *Pestoides F and *Synechococcus *sp. PCC 7002) to demonstrate the rapid manner in which mis-annotations can be found and explored in VESPA using either proteomics data alone, or in combination with transcriptomic data.

**Conclusions:**

VESPA is an interactive visual analytics tool that integrates high-throughput data into a genomic context to facilitate the discovery of structural mis-annotations in prokaryotic genomes. Data is evaluated via visual analysis across multiple levels of genomic resolution, linked searches and interaction with existing bioinformatics tools. We highlight the novel functionality of VESPA and core programming requirements for visualization of these large heterogeneous datasets for a client-side application. The software is freely available at https://www.biopilot.org/docs/Software/Vespa.php.

## Background

High throughput (HTP) molecular technologies are at the core of new capabilities to derive genomic-level profiles oforganisms [1,2]. One challenge often not addressed in the context of HTP technologies is the relationship of the analyses to the defined structural annotation of the genome. For example, the accuracy of global bottom-up proteomics is directly dependent upon accurately defined open reading frames (ORFs), because spectra are matched directly to an in silico enzymatic digest of the predicted proteins. Although a well-annotated genome is typically needed to analyze HTP data, it is also true that HTP data can contribute to genome annotation. Specifically, both next-generation sequencing transcriptomic data (RNA-Seq) and tandem mass spectrometry (MS/MS)-based proteomics have demonstrated immense value to genome curators [3-9] to locate features such as missed genes and intron/exon borders. While the procedural aspects of genome sequencing and assembly have become relatively inexpensive, the full and accurate annotation of these genomes, and integration of HTP data types to improve structural genome annotation is not straightforward, still very labor-intensive, and few computational tools have been developed to address this issue.

The development of RNA-Seq has been a major leap forward for transcriptomics, providing data to identify differentially expressed genes, as well as improve structural gene annotation. Common tools to process RNA-Seq data, such as IGV [10], SAMtools [11], Tablet [12], and Bambino [13], focus on aligning individual reads with the genome, because the number of reads aligned with particular genes can be used as a metric to quantify differential gene expression within the context of an experiment. Although most RNA-Seq experiments are focused on differential expression, the expression pattern in the context of the genome can yield information about transcriptional units, such as operons, and annotation errors, such as missed genes. Similar observations can also be made from other transcriptomics platforms, such as tiled arrays. However, visualization and analysis of these data in genomic context, in order to enhance the annotations or make inferences about mis-annotations, remains a challenge.

While transcriptomics data can give valuable insight into genome annotation, transcription does not necessarily mean translation into protein. Mass spectrometry-based proteomics can fill this gap through global identification of proteins expressed in a sample. However, similar to transcriptomics, proteomics usually focuses on comparative studies to identify differentially expressed proteins. In particular, in tandem mass spectrometry (MS/MS), spectra from proteolytic peptides are matched to theoretical spectra derived from candidate peptides from a defined genome annotation. In this traditional manner, only peptides from an annotated gene will be identified. However, in theory, proteomics data includes spectra from any gene translated into protein. Thus, an alternate strategy is to match spectra against peptide candidates from any potential open reading frame between two stop codons in any of the six frames of the DNA - *proteogenomics*. Proteogenomics experiments have successfully corrected gene locations (start sites), located novel genes, and identified additional various mis-annotations, such as frameshifts [7,14]; however, because mass spectrometry-based proteogenomics analyses require investigation of large numbers of potential peptides relative to the standard analysis, parsing and visualizing this data is challenging. Current software tools for proteomics data primarily focus on the processes of peptide identification, quantification and statistical comparison [15-18], whereas for proteogenomics, prokaryotic genome browser tools such as ARTEMIS [19,20] or Gbrowse [21,22] have been used due to their ability to compare different gene annotation models. To use these genome browsing tools for proteomics requires significant data formatting on the side of the user, because peptide identifications must be put into a standard format, such as a general feature format (GFF). Furthermore, there is no simple way to search for locations of interest in the genome, such as peptides located outside the defined gene annotations.

We present a novel software platform for Visual Exploration and Statistics to Promote Annotation (VESPA). VESPA was developed as a specialized tool within an overarching tool suite focused on the visualization and statistical integration of multiple data sources in a genomic context. VESPA 1.1.1 is a client-side Java application focused on assisting scientists with the annotation of prokaryotic genomes through the integration of proteomics (peptide-centric) and transcriptomics (probe or RNA-Seq) data with current genome location coordinates. VESPA visualizes all potential reading frames in a genome and has the capability to browse and query the data to quickly identify regions of interest with respect to structural annotation (e.g., novel genes, frameshifts). A basic proteotypic peptide statistic called SVM Technique to Evaluate Proteotypic Peptides (STEPP) [23] can be computed within VESPA, and used to filter peptides displayed in the visualization and queries. In addition, sequences of interest can be sent directly to BLAST [24] to assess the homology of genes identified within VESPA to known genes in the public databases. Alternatively, information extracted from the data, based on user queries to locate regions of interest, can be exported in easy-to-use formats for continued exploration outside of VESPA. VESPA is freely available at https://www.biopilot.org/docs/Software/Vespa.php. Here, we demonstrate the capabilities of VESPA with several use-case scenarios.

## Implementation

There are two modules in VESPA, an independent data analyzer module and the user interface (UI) platform. These two modules are installed together as one application built entirely in Java including an embedded H2 database http://www.h2database.com. From the data import and analytical operations, performance gains were obtained by a fast database running in embedded mode and by modularizing technical analysis of different types of data in several phases. Most of the intensive processing is performed at project creation or load so that quick data retrievals are possible.

The VESPA user interface is built using the Netbeans Platform and relies heavily on Java 2D for its visualizations http://netbeans.org/features/platform. Each visual component was built by extending existing Swing components for containers and providing custom paint code to render data from specific UI rendering models. The NetBeans platform provides a mature windowing system, module loader, persistence mechanism, and a Service Provider Application Programming Interface (API). With the Service Provider API, we developed a custom extension point or Service Interface and a Service Provider implementation that wraps the Analyzer. Using this platform, the visualization modules can be dynamically registered on start-up. This approach allows new visualizations to be added to the software with relative ease and additionally allows for an "auto update" feature that will download updates and new modules without any additional installation steps.

The VESPA Data Analyzer is written to read and process the various data types for storage in an H2 database. It relies heavily on the Apache POI libraries http://poi.apache.org to handle reading and writing Excel files. The Analyzer is independent from the UI, so that it can be used to process data independently or to load projects behind the scenes. After processing and storing the data the Analyzer serves up objects for the UI to visualize and query against. In-memory UI rendering models are used to provide a rapid response UI during navigation and trivial data filtering or searching. More complex searches and data exports are deferred to the database.

## Results

### Data import, processing and summarization

**Figure 1:**
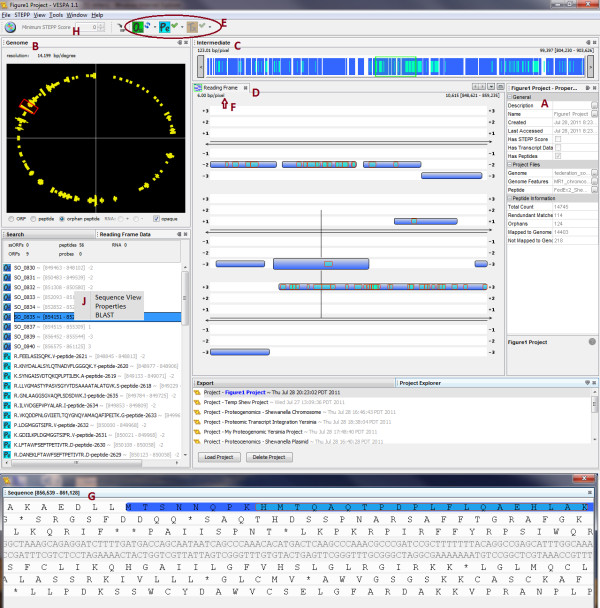
**Basic visualization and functional components of VESPA**. The default layout of VESPA displays: (A) the Project properties, (B) the Genome view with density of orphan peptides, (C) the Intermediate view, (D) the Reading Frame view, (E) the data select buttons, (F) the number of base pairs per pixel, which can be set by the user, (G) the Sequence view, (H) the STEPP threshold selector, and (J) the BLAST launch mechanism.

VESPA works under the concept of a project which, when created, at minimum requires the genomic sequence of a chromosome or plasmid (in FASTA format) and the defined gene features (ORFs and RNA genes in GFF format). Proteomics data may be provided in an Excel, csv or txt file, with at minimum two columns: one that contains the observed peptide sequences and a second that has an identifier for each peptide. Currently, formats such as pepXML are not supported, but many convertors to Excel are available [25]. VESPA supports two types of transcriptomic data: probes or RNA-Seq. Probes are imported in an analogous manner to peptides, with a single column for an identifier and a single column for the probe sequences. RNA-Seq data is imported as either a single Sequence Alignment/Map (SAM) file, or two Wiggle (WIG) files (positive and negative DNA strands) in which an observed count value is given for every genome coordinate location. Upon the completion of data processing and project creation, a summary panel (Figure 1A) summarizes the components of the project in terms of each imported file. For organisms with multiple genetic elements, a unique project can be created and saved for each element using the same proteomics file and transcriptomic files tailored to each DNA file. Once projects are created, visualization of the data in the context of each DNA element can be quickly achieved using the "Load Project" button.

### Visualization components

VESPA displays four levels of genome resolution, shown in the default layout in Figure 1. The highest level of resolution is the Genome View (Figure 1B), which shows an entire chromosome or plasmid, on which the density of features, such as orphan peptides or RNA-Seq, can be displayed. The second level of resolution is the Intermediate View (Figure 1C) along the top of the visualization; this view allows the user to more easily navigate around the Reading Frame View (third tier of resolution). The Reading Frame View (Figure 1D) is the primary visualization, displaying the double stranded DNA as thin black lines, with the three positive reading frames above and the three negative reading frames below these lines. This view wraps from left to right, and thus more of the genome can be visualized than in a single linear view. The proteomic and transcriptomic data are displayed directly within the Reading Frame View, and can be viewed or hidden using the buttons on the top control panel (Figure 1E). The resolution level of this screen can be modified by simply clicking on the "Number of Base Pairs per Pixel" and setting it to a desired level (Figure 1F). The fourth level of resolution is displayed based on regions defined by the user; a click and drag activity opens the Sequence View (Figure 1G), where all of the specific nucleic and amino acids in that region are displayed and accordingly color-coded to match the Reading Frame View. All individual visualization components can be easily resized within the application or completely un-docked from the application main window to allow the user to customize the application to suit their analysis task. The user-defined settings will be restored each time the application is launched, although, under the "Windows" pull-down selection the user may "Reset Windows" to the default view.

### Filter, query and export capabilities

**Figure 2:**
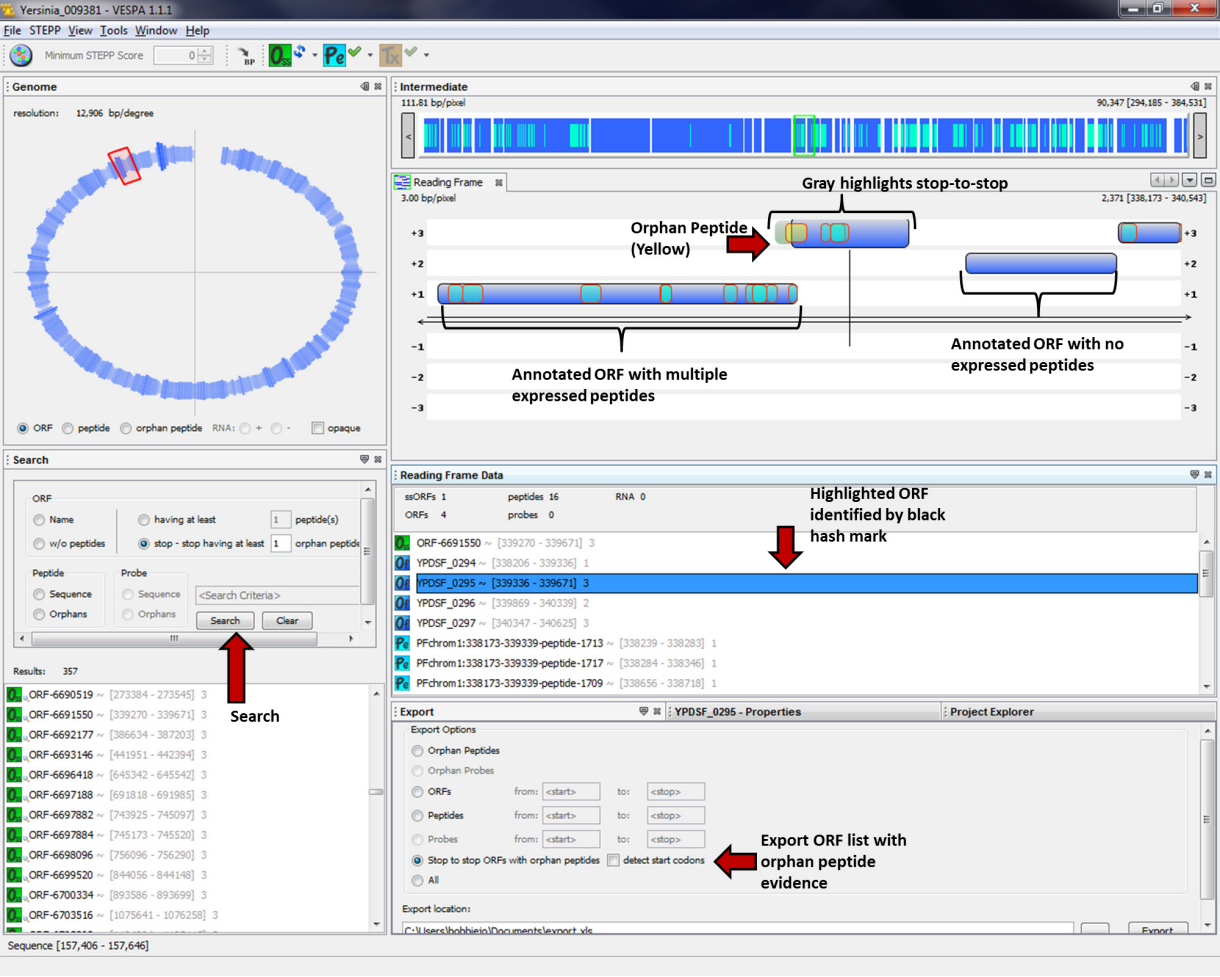
**Proteogenomic query**. VESPA evaluates the imported peptides against all potential ORFs in the genome. A search quickly highlights regions of interest with at least two orphan peptides; these can be exported for further analyses. This example shows a potential mis-annotation of the start site of an ORF in the *Y. pestis *pestoides F genome (accession CP000668).

The primary task of VESPA is to allow the user to quickly identify regions of interest in the genome without scrolling through millions of basepairs. The basic query interface of VESPA facilitates this, permitting targeted searches, such as for a specific gene (locus tag) or sequence (peptide or DNA), or more general searches, such as peptides that are not associated with genes in the current annotation (provided in the associated GFF file). These are termed orphan peptides, and are highlighted in yellow in the Reading Frame View, whereas peptides that are associated with an annotated gene are highlighted in light blue on that gene (dark blue). The region between two stop codons (*O_SS_*) encompassing a potential open reading frame that could have produced a particular orphan peptide appears highlighted in light gray to give the user an intuitive feel regarding whether the orphan peptide indicates the potential mis-annotation of a unique open reading frame or an extension of an annotated gene (i.e., a missed start). Figure 2 displays the result of a query to identify any *O_SS _*regions that have at least two orphan peptides. Queries are currently peptide-specific, although orphan probes can be displayed and RNA-Seq data can be filtered based on a minimum count threshold. Peptides can also be filtered based on the proteotypic peptide probability score defined by STEPP [23]. This probability score gives an estimation of the likelihood of observing a peptide by MS-based proteomics based on the peptide amino acid sequence composition (e.g., hydrophobicity, number of charged residues) (Figure 1H).

Users can export all orphan peptide locations and potential ORFs that meet query definitions (highlighted in Figure 2), or simple lists of ORFs, peptides or probes with coordinates. Furthermore, from within VESPA, a protein sequence of interest can be used as a BLAST query to search for sequence homology with proteins in the public databases at NCBI, by a right click on the protein in the Search or Reading Frame Data panel (Figure 1J). This gives the user a quick method to infer if an *O_SS _*of interest has homology with an annotated gene from another species, and could potentially be a true ORF.

## Discussion

### Simplified annotation discoveries through proteogenomic queries (case study 1)

Proteogenomics is focused on the utilization of MS/MS data to facilitate structural annotation efforts. VESPA dramatically simplifies proteogenomic tasks by allowing easy upload, browse, query and export capabilities for genomic and proteomic data. To demonstrate these capabilities, Figure 2 shows a screenshot of VESPA displaying proteomics data from *Yersinia pestis *Pestoides F. The project creation took in the genome files downloaded from GenBank (NC_009381.fna and NC_009381.gff) and an Excel table containing over 20,000 peptides, which were collected from a peptide identification search against protein translations of all potential coding regions in the *Y. pestis *Pestoides F genome. VESPA imported these peptides as their raw amino acid sequences, matching each peptide against all potential reading frames in the genome, thus not requiring prior mapping of the peptides to genomic coordinates.

**Figure 3:**
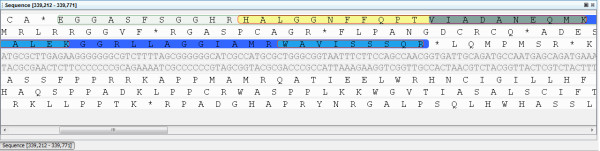
**Sequence view of orphan peptides and mis-annotated start sites**. Sequence view of the region highlighted in Figure 2. Here the specific orphan peptides (yellow) can be observed on the potential ORF circled in the +3 frame.

In the *Y. pestis *Pestoides F GenBank file, there are 3849 ORFs with annotated start and stop positions, however, there are 395,685 potential ORFs (*O_SS_*). Of the peptides identified against these 395,685 translated ORFs, there were 408 orphan peptides, peptides not fully contained within one of the 3849 annotated ORFs. A query for *O_SS _*with at least two orphan peptides identified 20 regions, shown in the Search results panel in Figure 2. These regions can be easily exported to an Excel file, using the export capability shown in the bottom right panel in Figure 2. In the Reading Frame View, the defined coding regions are highlighted in dark blue and regions that are *O_SS _*with orphan peptides associated with them are highlighted in light gray. All stop-to-stop regions can be observed by clicking on the green *O_SS _*button in the top control bar. Clicking on an *O_SS _*name in the results panel will center the visualization on that region. In the example shown, the observed orphan peptides (shown in yellow) are upstream and in the same frame as an annotated coding region for which many peptides were observed (shown in light blue). A drag and click across this region brings up the sequence view (Figure 3), from which the specific peptide sequences and underlying DNA sequence can be examined. This action reveals that there are no stop codons in the +3 frame in this region, suggesting that the start location of this ORF (YPDSF_0295) was mis-annotated. The locus tag and associated information for this gene, or for any feature in the visualization, can be viewed by simply clicking on the visualization feature to view the properties tab (tab next to the export tab on the bottom of Figure 2).

### Integrated omic data queries: Simultaneous evaluation of proteogenomic and transcriptomic data (case study 2)

**Figure 4:**
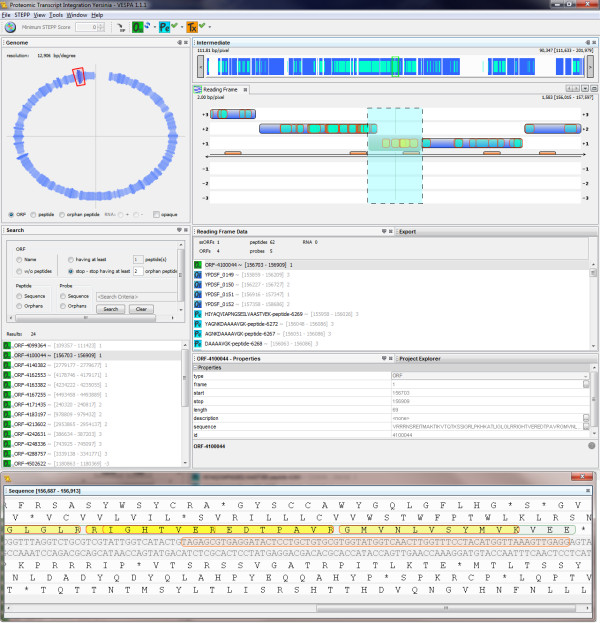
**Integration of probe-level transcriptomic data**. VESPA screenshot with probe data imported and layered into the proteogenomic view of *Y. pestis *Pestoides F. The imported probes are displayed on the DNA as orange rectangles. This region shows a potential unannotated ORF supported by 6 orphan peptides and 1 probe. The sequence view (bottom) simultaneously highlights the nucleic and amino acid sequences of the probe and peptides, respectively.

VESPA has been designed to integrate transcriptomics data into the visualization in the form of either defined probes (e.g., as from a microarray) or RNA-Seq. Probe data are imported as sequences in a similar manner to the proteomics import. All probes are drawn as defined by the sequence data in the input file, and are viewed on the DNA strands as orange rectangles over the sequence region (Figure 4). The user can identify orphan probes or locate probes by sequence, but since probes are not associated with a defined frame they are currently not linked to *O_SS_*. RNA-Seq data are imported as either two WIG files, with coordinates and coverage values for the positive and negative DNA strands, or as a SAM file from which the coordinates and coverage values are computed. By default, the coverage values are displayed in the Reading Frame view as an orange histogram of the log of the coverage values. Specific values are not shown, and the highest value is set to visually reach the edge of the +/-3 reading frame, and all other intensity values are scaled with respect to this maximum. VESPA is most functional with both proteomics and transcriptomics data, however, it can be utilized with only transcriptomics data. Current queries for interesting *O_SS _*regions are peptide-centric and thus functionality without proteomic data is limited, but a topic of further development.

#### Integration of proteomics and probe-based transcriptomic data

**Figure 5:**
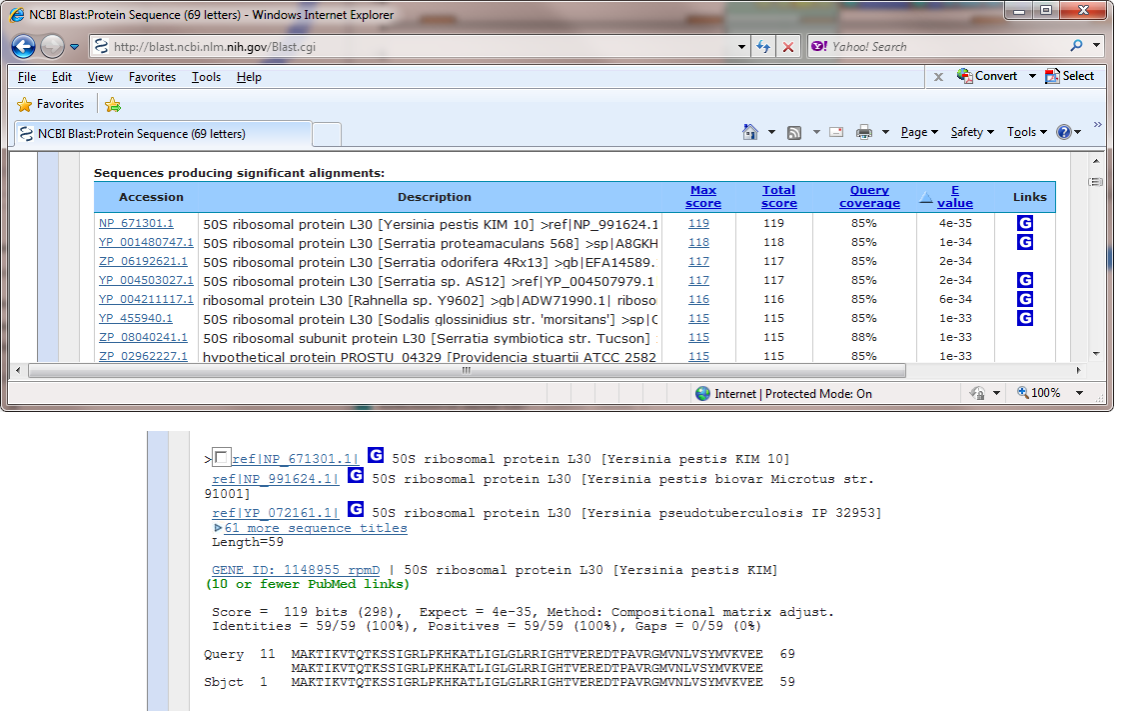
**BLAST query of missed ORF in *Y. pestis***. Screenshots of the NCBI BLAST results page from a basic protein-protein search against the NR database for ORF-4100044 (Figure 4) show clear homology to annotated proteins in related Yersiniae.

In addition to the proteomics data, microarray probe data were collected for *Y. pestis *Pestoides F [26]. There were 6333 probes with expression values above the defined threshold (signal intensity ≥ 25,000): 4975 overlapped completely with one of the 3849 annotated ORFs, and 1358 were outside any annotated ORF boundaries. Figure 4 shows the VESPA Reading Frame and Sequence Views for an example of a missed (unannotated) ORF between YPDSF_150 and YPDSF_151, for which 6 unique peptides and 1 unique probe were identified. The probe data provides evidence of transcription and the peptides confirm the presence of a protein encoded in the +1 frame of this region. The "Reading Frame Data" panel shows the names associated with each feature viewed in the Reading Frame View; a right click on the green *O_SS _*ORF-410044 launches BLAST, the results of which show high homology to a 50 S ribosomal protein from other *Yersinia *species (Figure 5). Specifically, starting at residue 11 (the likely start position) of the *O_SS_*, this short protein is an identical match to the L30 ribosomal protein in a number of *Y. pestis *strains.

#### Integration of proteomics and RNA-Seq transcriptomic data

**Figure 6:**
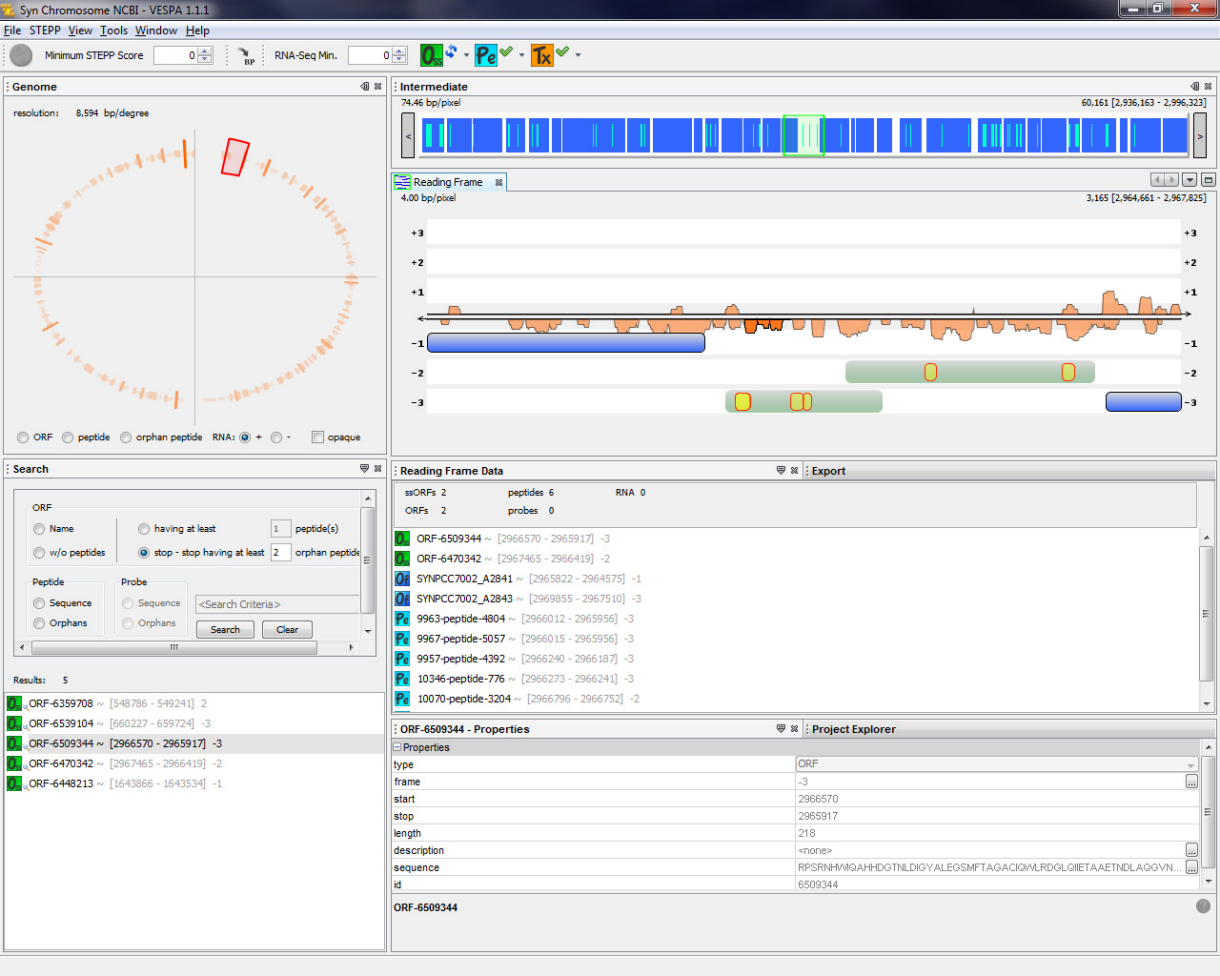
**Integration of RNA-Seq transcriptomic data**. VESPA screenshot with RNA-Seq data layered into the proteogenomic visualization of *Synechococcus *sp. PCC 7002, showing expression of the annotated pseudogene SYNPCC7002_A2842.

**Figure 7:**
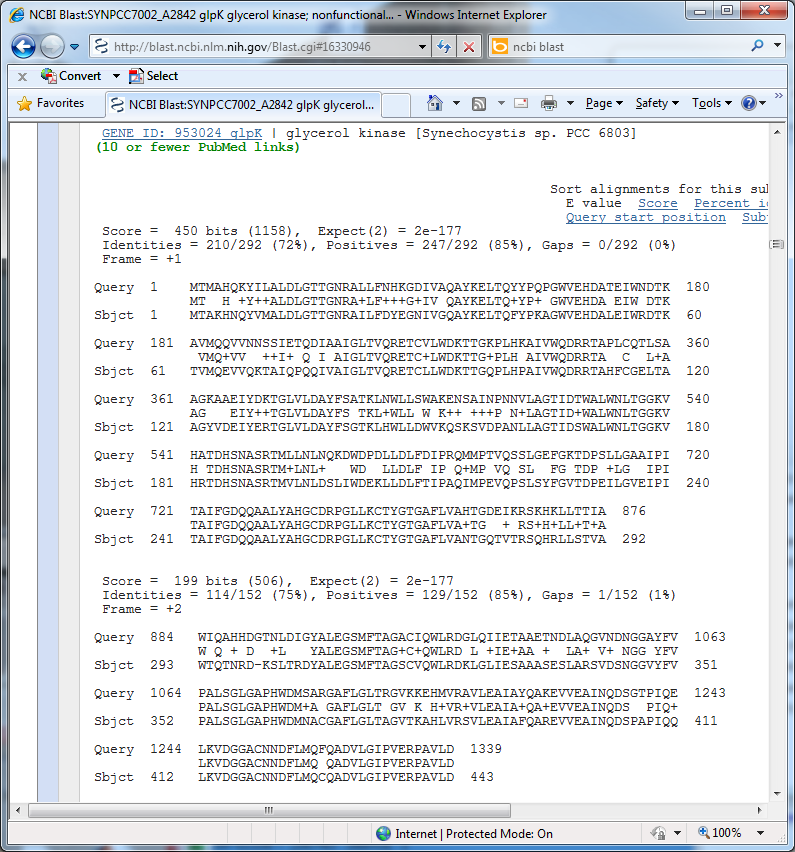
**BLAST of *Synechococcus *sp**. PCC 7002 pseudogene. Screenshots of the NCBI BLAST results page from a blastx search, searching the NR protein database with the translated DNA sequence of the pseudogene SYNPCC7002_A2842, shows the frameshift in the query necessary to return high homology matches to the annotated glycerol kinase of *Synechocystis *sp. PCC 6803.

To demonstrate VESPA's integration of RNA-Seq data, we examined the chromosome of *Synechococcus *sp. PCC 7002 (accession NC_010475), which has 2824 defined ORFs and 224,169 *O_SS _*regions. To create this project, genome files from RefSeq were imported (NC_010475.fna and NC_010475.gff), together with an Excel table of 6016 peptides identified by matching the proteomics spectra to the protein translations from the *O_SS _*regions for all 7 genetic elements (one chromosome and six plasmids) for this organism [27], and a SAM file of RNA-Seq coverage values for the chromosome [28]. VESPA identified 5398 peptides that map to the annotated ORFs and 364 orphan peptides, show in yellow in Figure 6. The RNA-Seq data are shown in the visualization as an orange histogram of the log of the coverage value at each position. Figure 6 shows an example in which two neighboring *O_SS _*regions in the -2 and -3 frames, between SYNPCC7002_A2841 and SYNPCC7002_A2843, have peptide evidence from several observed peptides and RNA-Seq data observed on the negative strand along this entire span. Examination of the genome feature file (NC_010475.gff) reveals that these two *O_SS _*regions belong to SYNPCC7002_A2842, which is annotated as a pseudogene; specifically, the glycerol kinase gene rendered non-functional by a frameshift. A further evaluation of this region via BLAST confirms that both *O_SS _*regions have high homology to other cyanobacterial glycerol kinases that are intact (Figure 7). While it is possible that an intact SYNPCC7002_A2842 protein could be translated from the annotated gene by a mechanism such as ribosomal slippage, most known cases of translational frameshifting in prokaryotes are insertion sequence or phage genes [29]. Thus the frameshift in SYNPCC7002_A2842 is more likely the result of an error in the genome sequence; resequencing of this region of the *Synechococcus *sp. PCC 7002 genome has in fact revealed errors in the original DNA sequence, the correction of which results in an intact SYNPCC7002_A2842 gene (D. Bryant, unpublished observations).

**Figure 8:**
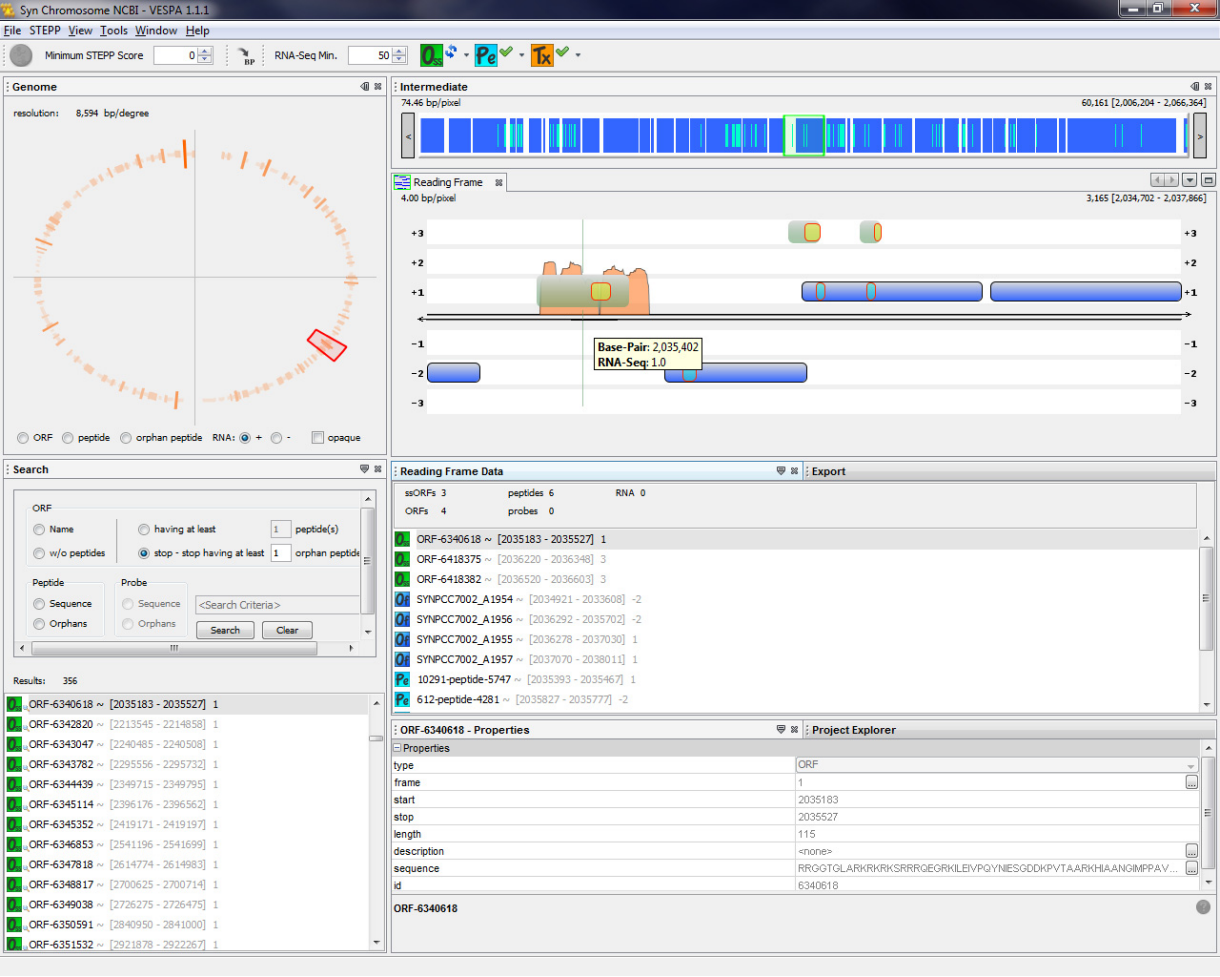
**RNA-Seq data filtering**. VESPA screenshot of an *O_SS _*region detected with a single orphan peptide identification and the RNA-Seq data filtered to require a minimum log(coverage value) of 50.

We also used the *Synechococcus *sp. PCC 7002 data to demonstrate the value of RNA-Seq data viewed in combination with weak peptide data. Specifically, in the *Synechococcus *sp. PCC 7002 proteomics data there are five *O_SS _*regions with at least two orphan peptides, and an additional 351 *O_SS _*regions with only one orphan peptide. Single orphan peptides are often false identifications and thus dismissed without further investigation, however in these cases, RNA-Seq can be very useful to separate true from false peptide identifications. Figure 8 shows an example of an *O_SS _*region in the +1 frame with a single identified peptide. Using VESPA to view the RNA-Seq data in this *O_SS _*region with a filter requiring log(coverage value) > 50 shows a clear expression pattern, supporting the idea that a missed ORF is coded in this region. A BLAST search for homologs to this *O_SS _*identifies a putative conserved domain (DUF3155 superfamily) and 59 sequences in the NCBI nr database with significant alignments (E-value < 1e-10). In this case, all the significant alignments were to a hypothetical protein, and the highest scoring alignment was to a protein from *Nostoc azollae *0708 (data not shown).

## Conclusions

Despite recent advances in the generation and processing of high-throughput proteomics and transcriptomics data, the availability of visual analytics tools for the purposes of studying genome annotation, as well as the integration and exploration of these data streams in concert, remains a challenge. Here we have presented VESPA, a freely available software tool, for the purpose of proteogenomics and the integration of peptide-centric data with other forms of high-throughput transcriptomics data. The proteogenomic queries available through VESPA enable the discovery of regions of mis-annotations in a rapid manner, which can reduce the number of candidate reading frames for evaluation.

While VESPA and similar software tools facilitate data integration to improve genome annotations, the contribution of annotation corrections back to the genome databases is an ongoing challenge within the genomics community. NCBI does provide a mechanism to submit Third Party Annotations based on experimental evidence http://www.ncbi.nlm.nih.gov/genbank/tpa. Though these annotations do not become incorporated into the whole genome annotation unless the genome project owner updates the genome, this at least provides a mechanism to make specific gene annotation corrections found using VESPA publicly available.

VESPA is designed with a plug-in-play architecture to allow the addition the new visualizations and query interfaces. Future development will include the enhancement of these capabilities, such as the ability to query and filter on transcriptomic data and the visualization of CHIP-Seq data. In addition, VESPA enhancements are planned that will allow ingest of additional data formats (e.g., GenBank, pepXML), and facilitate the analysis of genomes with multiple genetic elements. Since VESPA has an auto-update feature that will notify the user when newer versions are available, the user will have nearly immediate access to these features as they are added.

### Methods - biological case studies

#### Synechococcus sp. PC 7002

The *Synechococcus *sp. PCC 7002 data generation methods have been described previously [27,28], thus the data generation and analyses will be only briefly described here. The proteomics and RNA-Seq experiments were performed on cells grown under atmospheric CO_2 _levels to study photorespiration processes in this organism.

##### LC-MS/MS

The *Synechococcus *sp. PCC 7002 cell samples were processed for LC-MS/MS proteomics essentially as described previously [27]; the peptide identification computations were performed at the Molecular Sciences Computing Facility at the Environmental Molecular Sciences Laboratory (Richland, WA). MS/MS peaks were determined using DeconMSn, v2.2 [30] and MSPolygraph [31] was used for identifying peptides. Tryptic peptides were searched for using a parent mass-to-charge window of +/- 3 Da, and fragment ion windows of +/- 1 Da. Two missed cleavages were allowed for peptides with a parent mass charge of +1, three for +2 parent mass peptides, and four missed cleavages for peptides with a parent mass of +3. The spectra were searched against a six frame translation (no minimum length was imposed) of the *Synechococcus *sp. PCC 7002 genome and plasmids (NC_010474 through NC_010480) and spectra matching peptides at least six amino acids in length reported. For estimating error rates, random peptides were generated using the program *mimic*, released with *percolator *[32]. The false discovery rates (q-values) were estimated with *qvality *[33]. Peptides identified at a q-value < 5% were retained for visualization in VESPA.

##### RNA-Seq

The *Synechococcus *sp. PCC 7002 RNA-Seq data were generated from an 0.5 μg RNA sample using a SOLiD™ Whole Transcriptome Analysis Kit (Applied Biosystems) and the SOLiD™ 3Plus protocol as described previously [28]. Sequencing was performed at the Genomics Core Facility at The Pennsylvania State University (University Park, PA). The raw RNA-Seq data were processed as described previously [28], the sequence reads mapping to rRNA-coding regions removed and a SAM file generated for VESPA import.

#### Yersinia pestis pestoides F

The *Y. pestis *Pestoides F data were a subset of data collected for a larger experiment focused on the comparison of genome annotations across multiple Yersiniae strains [26]. Here we briefly describe the methods used to generate the proteomics and global microarray data.

##### LC-MS/MS

Peptides (0.5 μg/μL) from global preparations (run in triplicate, total of n = 30 LC-MS/MS runs per strain), and SCX fractionated samples (n = 48 fractionated samples run per strain) were separated by a custom-built nanocapillary HPLC system. The eluate from the global preparations and fractionated samples was directly analyzed by electrospray ionization (ESI) using a LTQ Orbitrap Velos mass spectrometer or linear ion trap (LTQ) mass spectrometer (Thermo Scientific), respectively. Raw data are available to the public at http://omics.pnl.gov. MS/MS fragmentation spectra were searched against a six frame translation (minimum open reading frame length of 30 amino acids) of *Y. pestis *Pestoides F genome and plasmids using the SEQUEST peptide identification software [34]. The mass tolerance used for matching was set to ± 3 Da. Peptide identifications were retained based upon the following criteria: 1) SEQUEST DelCn2 value ≥ 0.10; 2) SEQUEST correlation score (Xcorr) ≥ 1.9 for charge state 1+ for fully tryptic peptides and Xcorr ≥ 2.20 for 1+ for partially tryptic peptides; Xcorr ≥ 2.2 for charge state 2+ and fully tryptic peptides and Xcorr ≥ 3.3 for charge state 2+ and partially tryptic peptides; Xcorr ≥ 3.3 for charge state 3+ and fully tryptic peptides and Xcorr ≥ 4.0 for charge state 3+ and partially tryptic peptides. Using the reverse database approach, the false discovery rate (FDR) was calculated to be < 0.4% at the spectrum level.

##### Universal Yersinia Microarray

The global microarray data included 7641 designed oligos from *Y. pestis *strains CO92, KIM, Pestoides F, Antiqua, Nepal516, and biovar Microtus str. 91001, and *Y. pseudotuberculosis*. The array platform description and oligo list is available at NCBI Gene Expresssion Omnibus (GEO) under accession GPL9009. Scanning, image analysis, and normalization were performed as outlined in PFGRC standard protocol http://pfgrc.jcvi.org/index.php/microarray/protocols.html. Individual TIFF images from each channel were analyzed with JCVI Spotfinder software (available at http://pfgrc.jcvi.org/index.php/bioinformatics.html). Microarray data were normalized by LOWESS normalization using TM4 software MIDAS http://pfgrc.jcvi.org/index.php/bioinformatics.html. Oligos generating intensity signals ≥ 25,000 from samples prepared at 1 hour time point under 37 degree growth were considered to have positive hybridization above background and therefore incorporated as experimental measurements. Transcriptomics data have been deposited in the GEO repository under series accession GSE30634.

## Availability and requirements

VESPA is freely available at https://www.biopilot.org/docs/Software/Vespa.php with installers for Windows XP, Windows 7, Macintosh and Linux. Java Runtime Environment 1.6 is required to run the application.

## Abbreviations

SOLiD: Sequencing by Oligonucleotide Ligation and Detection; GFF: General Feature Format; BLAST: Basic Local Alignment Search Tool; API: Application Programming Interface; UI: User Interface

## Competing interests

The authors declare that they have no competing interests.

## Authors' contributions

BMW, ESP and LAM conceived the approach; ESP, JLJ MAK and HW developed the software. ACR, LAM, SRW, SHP, CKA, WRC and JNA identified biological case studies and assembled the appropriate files. BMW, LAM and ESP wrote the manuscript and all authors read and approved of the final manuscript.
